# Acute exercise does not modify brain activity and memory performance in APP/PS1 mice

**DOI:** 10.1371/journal.pone.0178247

**Published:** 2017-05-22

**Authors:** Angelica Miki Stein, Victor Munive, Ana M. Fernandez, Angel Nuñez, Ignacio Torres Aleman

**Affiliations:** 1 Cajal Institute, Madrid, Spain; 2 Ciberned, Madrid, Spain; 3 Universidade Estadual Paulista, São Paulo, Brazil; 4 School of Medicine, Autonoma University of Madrid. Madrid, Spain; University of Lethbridge, CANADA

## Abstract

Age is the main risk factor for Alzheimer´s disease (AD). With an increasingly aging population, development of affordable screening techniques to determine cognitive status will help identify population-at-risk for further follow-up. Because physical exercise is known to modulate cognitive performance, we used it as a functional test of cognitive health. Mice were submitted to treadmill running at moderate speed for 30 min, and their brain activity was monitored before and after exercise using electrocorticogram (ECG) recordings. After exercise, normal, but not APP/PS1 mice, a well established AD model, showed significantly increased ECG theta rhythm. At the same time normal, but not AD mice, showed significantly enhanced performance in a spatial memory test after exercise. Therefore, we postulate that a running bout coupled to pre- and post-exercise brain activity recordings will help identify individuals with cognitive alterations, by determining the presence or absence of exercise-specific changes in brain activity. Work in humans using a bout of moderate exercise plus electroencephalography, a clinically affordable procedure, is warranted.

## Introduction

With an increasingly larger proportion of aged people in modern societies, cognitive loss in the aging population is becoming a paramount problem for public health agencies. Hence, an urgent need for early identification of individuals at risk of developing cognitive loss is widely recognized [1]. Current clinical practice to determine the presence of cognitive problems relies entirely on psychometric tests that are reliable but laborious, and require well-trained personnel [2, 3]. Furthermore, these tests are useful to detect early signs of cognitive disturbances, already when deficits are present [3]. Intense focus on this problem has resulted in the appearance of new diagnostic techniques based on biochemical parameters in cerebro-spinal fluid (CSF) and in brain imaging. The first approach is problematic because CSF samples are not easy to obtain. The second one is expensive and requires state-of-the-art facilities. In both cases, highly trained professionals are a must. On top of that, any of these procedures have yet been implemented for early diagnosis, prior to the appearance of cognitive disturbances.

As exemplified by cardiovascular screening programs, preventive screening procedures of the general population are an optimal way to detect populations-at-risk, but difficult to support with available diagnostic procedures even by public health systems of developed countries. Therefore, development of affordable and more objective tests would make much more feasible this goal. From this perspective, for the present study we chose clinical procedures of wide use such as a bout of exercise (for cardiovascular screening) and electroencephalographic recording (for neurological testing) and combined them to develop an easy-to-implement procedure. As a single bout of exercise has been shown to improve cognition both in humans [4, 5], and rodents [6], and physical fitness helps predict cognitive loss [7, 8], we reasoned that cognitive status could be determined using exercise as an stimulus.

At the same time, exercise acutely modulates brain activity in humans [9], and rodents [10]. Brain activity can be monitored by a number of different techniques, but electroencephalography (EEG) is probably the most extended and used. For many years, EEG recordings have been extensively applied to AD patients with the idea of using this technique as a diagnostic tool. Three major effects of AD on EEG have been observed: slowing of the EEG, reduced complexity of the EEG signals, and perturbations in EEG synchronization [11, 12]. A major disadvantage of this technique for AD diagnosis is that the EEG changes observed in AD patients are shared by other pathologies such as different types of dementia, mild cognitive impairment [13], and encephalopathy [14]. Moreover, EEG recordings have been performed in AD patients because AD is associated with an elevated risk for seizures. It is known that people with AD are 10 times more likely to develop epilepsy than the age-matched general population (for a review see [15]. However, only 1.5% of AD patients develop seizures. With the idea of gaining additional support to the use of EEG as a diagnostic procedure in AD, and based on these prior observations, we speculated that the pro-cognitive actions of acute exercise could be lost in AD patients and could be reflected in specific alterations in the EEG pattern. For this reason, and as an initial proof-of-concept experiment, we combined exercise with electrocorticographic recordings in control and APP/PS1 mice to determine possible differences between the two groups. The results show that an exercise-based test aids to identify cognitive deterioration using electrical activity recordings of the brain as a read-out.

## Materials and methods

### Animals

Male adult C57BL/6J mice (19–23 g, 4–6 months old; Harlan Laboratories, Spain) and in-bred APP/PS1 mice of the same age and genetic background [16] were housed in standard cages (48 × 26 cm^2^) with 5 animals per cage. Mice were kept in a room with controlled temperature (22°C) under a 12-12h light-dark cycle; fed with a pellet rodent diet and water ad libitum. All experimental protocols were performed during the light cycle. Animal procedures followed European guidelines (2010/63/EU) and were approved by the local Bioethics Committee (Madrid Government). The presence of brain amyloid deposits was confirmed by immunocytochemistry in APP/PS1 mice (S1A Fig).

### Electrocorticogram recordings in freely moving animals

Adult WT and APP/PS1 mice were anesthetized with isofluorane (2–3% for induction) mixed with O2 (0.5–1 L/min) and placed in a stereotaxic device. The skin was cut along midline and a craniotomy was made (0.5 mm diameter) on the parietal cortex (AP: -2, L: 4: V: 1 mm, from the bone surface). A stainless steel macro-electrode of <0.5 MOhms was placed without disrupting the meninges to register the electrical cortical activity (ECG), using a DSI Implantable Telemetry device (Data Sciences International). After surgery to implant the transmitter device, mice remain in their cages a minimum of 4 days to recover. Animals were then placed in the treadmill chamber 15 or 30 minutes for familiarization. Animals were recorded after familiarization. ECG baseline was registered during 5 minutes (pre-running control period) and another 5 minutes immediately after running. Animals run 15 minutes the first two days and 30 minutes the following 2 days. Signals were stored in a PC using DSI software and filtered off-line between 0.3–50 Hz with Spike 2 software (Cambridge Electronic Design, Cambridge, UK). ECG segments of 5 minutes were analyzed by Spike 2 software, using the Fast Fourier Transform algorithm to obtain the power spectra. The mean power density was calculated for 5 different frequency bands that constitute the global EEG: delta band (0.3–4 Hz), theta band (4–8 Hz), alpha band (8–12 Hz), beta band (12–30 Hz) and gamma band (30–50 Hz). The total power of the five frequency bands were considered 100%, and the percentage of each frequency band was calculated.

### Recordings in anesthetized animals

Experiments were performed on 6 urethane anesthetized (1.6 g/kg ip) adult WT mice. Animals were placed in a Kopf stereotaxic device in which surgical procedures and recordings were performed. Supplemental doses of anesthetic were given to maintain areflexia. Local anesthetic (lidocaine 1%) was applied to all skin incisions and pressure points. An incision was made exposing the skull, and small holes were drilled in the skull over the parietal cortex (coordinate as above) and over the CA1 hippocampal area (AP: -1.5, L: 2: V: 2 mm). ECG and hippocampal field potential (HFP) were recorded with tungsten macroelectrodes (<1 MOhms World Precision Instruments, Sarasota, FL). Recordings were filtered (0.3–50 Hz), amplified via an AC preamplifier (DAM80; World Precision Instruments), and fed into a personal computer (sample rate 200 Hz) for off-line analysis with Spike 2 software. The mean power density was calculated from 5 minutes of spontaneous activity or when theta rhythm was evoked by sensory stimulation (stroking the fur on the animal’s back). The percentage of theta rhythm was calculated in the ECG and HFP. Cross-correlations between the theta rhythm recorded in the ECG and the HFP were calculated for 5 minutes of spontaneous activity or during sensory stimulation. Previously, recordings were digital filtered between 4–8 HZ (theta frequency band) to isolate the theta rhythm.

### Treadmill running

Before submitting animals to treadmill running, they were handled daily and familiarized with the apparatus (Letica, Italy) to minimize novelty stress. The electrical shock system that encourages the animals to run was disconnected to avoid pain stress. The exercise group ran for 15 and 30 min in separate days and at a moderate speed: the first two minutes speed was gradually increased to reach a final steady speed of 9 m/min. The control group remained for the same time in the treadmill without running. We chose this mild intensity exercise regime for two reasons: 1) to avoid changes in stress hormones that could interfere with post-exercise behavioral assessment and 2) to avoid fatigue as the protocol is intended to be translated into clinical testing. Additional groups of animals were used for behavioral testing (see below).

### Y-maze

Working memory was assessed by recording spontaneous exploring behaviour in a Y-maze [17]. The maze was made of black-painted wood and each arm was 25 cm long, 14 cm high, 5 cm wide and positioned at equal angles. Before treadmill running, and after the animals had remained in the treadmill apparatus for ~30 minutes to adapt to the novel environment, they were placed at the end of one of the arms of the maze and allowed to move freely during a 5 min session with one of the arms randomly blocked. Thereafter they were allowed to recover for 90 minutes before running for 30 minutes in the treadmill. After running, they were placed again in the maze with all three arms opened (see S1B Fig). Number of entries in the “new” arm were scored and compared to those to the “old” arms. The whole session was recorded by video and analyzed later using Ethovision. Arm entry was considered to be completed when the hind paws of the mouse were completely placed inside the arm. An alternative, shorter, inter-trial test of 60 min was also used in another group of animals to determine whether sedentary mice could learn a less demanding procedure.

#### Blood lactate

Lactate was measured in blood before and after running to determine exercise intensity. Blood was collected from the tail vein using a small puncture with a surgical knife. First drop of blood was discarded and the second one was used to determine lactate levels using a blood lactate analyzer and reagent strips by Lactate Plus® (Tanner, Fuller, Ross, 2012). Because the ECG device inserted on the head of the animals may cause physical alterations, we determine lactate both in sham operated and ECG-operated mice; lactate values were found to be similar and pooled together.

### Aβ immunocytochemistry

Immunocytochemical assays were run as described before [16]. Animals were deeply anesthetized with pentobarbital (50 mg/kg) and perfused transcardially with 4% paraformaldehyde in 0.1 M phosphate buffer, pH 7.4 (PB). Coronal 50-μm-thick brain sections were cut in a vibratome and collected in PBS. Sections were incubated with 100% methanol and 0.03% H_2_O_2_ to eliminate endogenous peroxidase followed by incubation overnight at 4°C with primary antibody in PB- 1% bovine albumin- 1% Triton X-100. For immunocytochemistry of Aß plaques, a pre-treatment of 70% formic acid was used before incubation with anti-human Aß antibody (1:50, Dako clone 6F/3D). After several washes in PB, sections were incubated with a biotin-coupled secondary antibody (1:500, Pierce) followed by ABC amplification system (1:250, Pierce) using diaminobenzidine as chromogen. Sections were dehydrated and mounted with DEPEX. Omission of primary antibody was used as control. Panoramic pictures were obtained with a Leica (Germany) microscope using the stitching tool.

### Statistics

Statistical analysis was performed using GraphPad Prism 5 software (San Diego, CA, USA) and SPSS. All results are shown as mean ± s.e.m. After normal distribution was confirmed using the Saphiro-Wilk test, we used the paired Student’s *t*-test when comparing pre- and post-exercise data, the *t*-test for independent variables when comparing the two experimental groups, and one-way analysis of variance followed by Bonferroni´s test when comparing multiple groups. Probability values <0.05 were considered significant.

## Results

### Exercise modifies electrocorticogram activity and improves cognitive function in control but not AD mice

**Fig 1:**
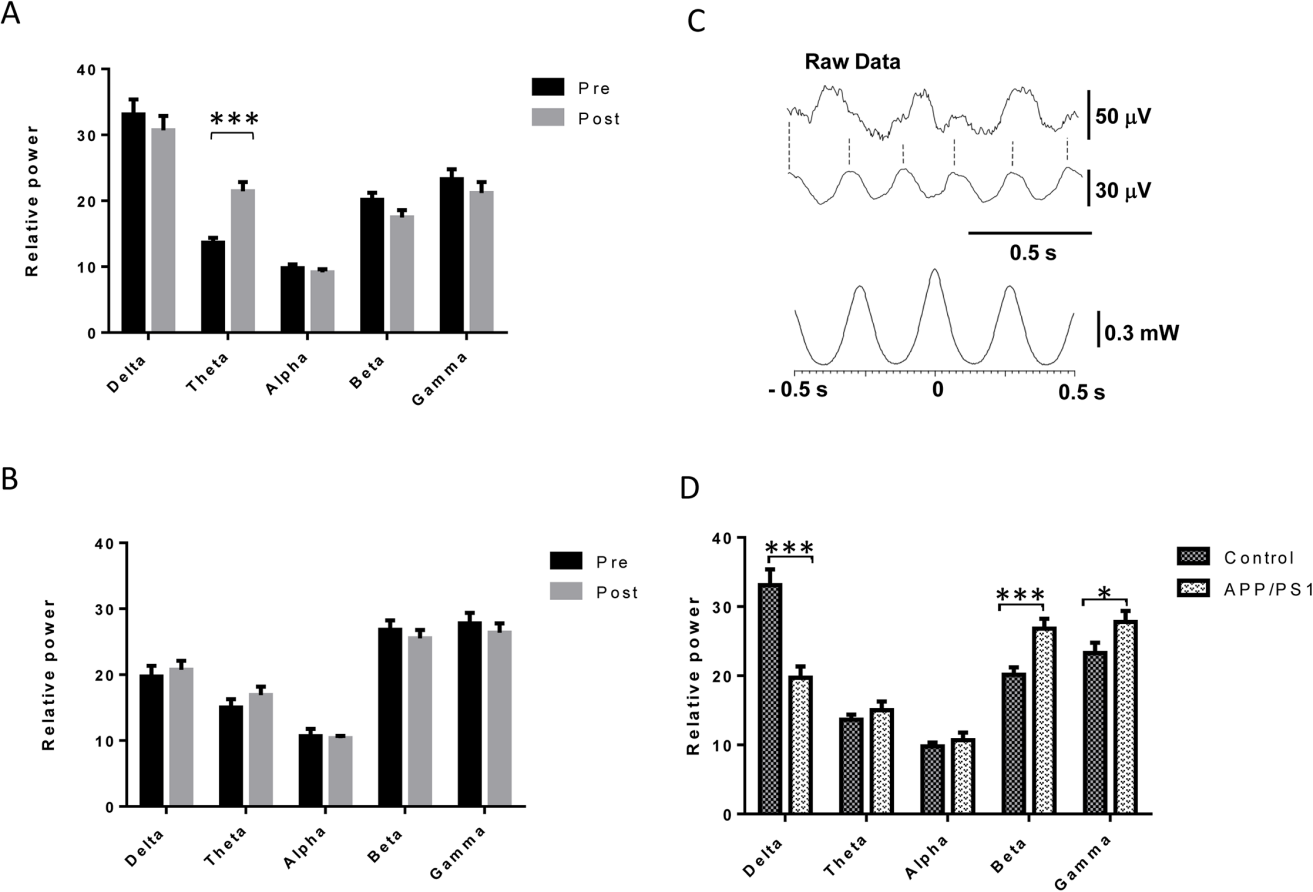
Differences in baseline and post-exercise electrocorticogram activity in APP/PS1 mice. **A,** A bout of moderate exercise specifically increases theta frequencies in the elecorticogram (ECG) of wild type mice (T = -5,72; df = 33; *p<0.05; n = 9). **B,** No changes are seen in APP/PS1 mice (n = 9) after exercise. Pre: 5 min ECG recordings before exercise; Post: 5 min ECG recordings after exercise. Results are the mean of pooling together ECG recordings performed by each mouse after 15 and 30 min of exercise in independent days. **C,** Electrophysiological simultaneous recordings of the ECG (upper record) and of the hippocampal CA1 region (lower record) when theta rhythm was evoked by sensory stimulation (Raw Data). Dashed vertical lines indicate the correspondence of positive peaks of hippocampal theta rhythm with positive peaks in the ECG. Lower plot shows the CC of the hippocampal theta rhythm respect to ECG activity after digital filtering between 4–8 Hz. Periodic peaks implies phase-locking of both theta rhythms. **D,** Baseline ECG recordings in wild type and APP/PS1 mice show differences in various frequency bands (Delta: t = 4,8; df = 68,56; Beta: t = -3,76; df = 72; Gamma: t = -2,04; df = 72; *p<0.05; ***p<0.001; n = 9 per group). Results shown are mean ± S.E.M.

Each animal run in independent sessions and in separate days for 15 and for 30 minutes, at moderate speed in the treadmill. For each running session, electrocorticogram recordings (ECG) were carried out in the parietal cortex in freely moving conditions before and after exercise. As shown in Fig 1, control, but not AD mice showed a significant increase in the theta frequency band of the ECG after exercise.

WT mice increased theta frequencies after 15 min or 30 min of exercise (13.8% in control to 22.7%, p<0.001 vs. pre-exercise; or to 18.8%, p<0.001, vs. pre-exercise, respectively). No statistical differences were observed between the percentage of frequency bands after 15 or 30 min of exercise and for this reason the results were pooled together in the figures. In contrast, APP/PS1 mice did not alter the proportion of frequency bands after 15 or 30 min of exercise. The power of the theta frequency band was 3.2 ± 1.2% (p>0.05, n = 9) and 2.1 ± 1.1% (p>0.05, n = 9) after 15 and 30 minutes of exercise, respectively.

In WT mice, exercise induced an increase of theta waves in the parietal ECG. The origin of theta waves may be the hippocampus where an increase of theta rhythm after exercise has been demonstrated [18]. To test if theta waves recorded in the ECG are generated in the hippocampus, ECG and hippocampal field potentials (HFP) were recorded simultaneously under control conditions and during sensory stimulation in urethane anesthetized WT mice (n = 6). In control conditions, theta rhythm represented 8.3% and 11.5% of the ECG and HFP, respectively. These percentages increased up to 15.5% and 20.9%, respectively, during sensory stimulation of the animal’s back. Raw data showed theta activity in the HFP during sensory stimulation while the ECG showed theta oscillations between larger delta waves evoked by the anesthetic (Fig 1C, Raw Data). Moreover, cross-correlation (CC) of theta rhythm recorded in the ECG and HFP showed that theta rhythms during sensory stimulation were in phase because the CC showed periodic peaks around the zero reference (Fig 1C, lower plot). Thus, the fact that both theta rhythms changed similarly in both conditions strongly suggests that the theta rhythm recorded in the parietal cortex reflect, at least in part, the hippocampal theta rhythm.

In control conditions (before exercise), WT mice (n = 9) showed a 34.4% of delta waves, 13.9% of theta, 9.7% of alpha, 19.4% of beta and 22.6% of gamma frequency bands. However, APP/PS1 mice (n = 9) showed a significant decrease of the percentage of delta waves (19.5%; p<0.001) and an increase of beta (26.9%; p<0.001) and gamma (27.9%; p = 0.04) frequencies. Theta and alpha frequencies did not change in APP/PS1 mice (15.0% and 10.7%, respectively) respect to values of WT animals, suggesting that the cortical activity is faster in APP/PS1 mice at rest (Fig 1D).

Interestingly, as reported in human patients [19], blood lactate levels in APP/PS1 mice were higher at rest, but did not increase after running, whereas in control mice a moderate increase was seen (Table 1).

**Table 1:** Blood lactate changes in wild type (WT) controls and AD (APP/PS1) mice after 15 and 30 minutes of moderate running exercise.

	Pre-exercise (mean ± sd)	15 min (mean ± sd)	30 min (mean ± sd)
**WT**	**1.92 ± 0.93**	**3.28 ± 0.86**	**2.77 ± 1.43**
**APP/PS1**	**2.61 ± 0.24**	**2.94 ± 0.25**	**2.72 ± 0.26**

**Fig 2:**
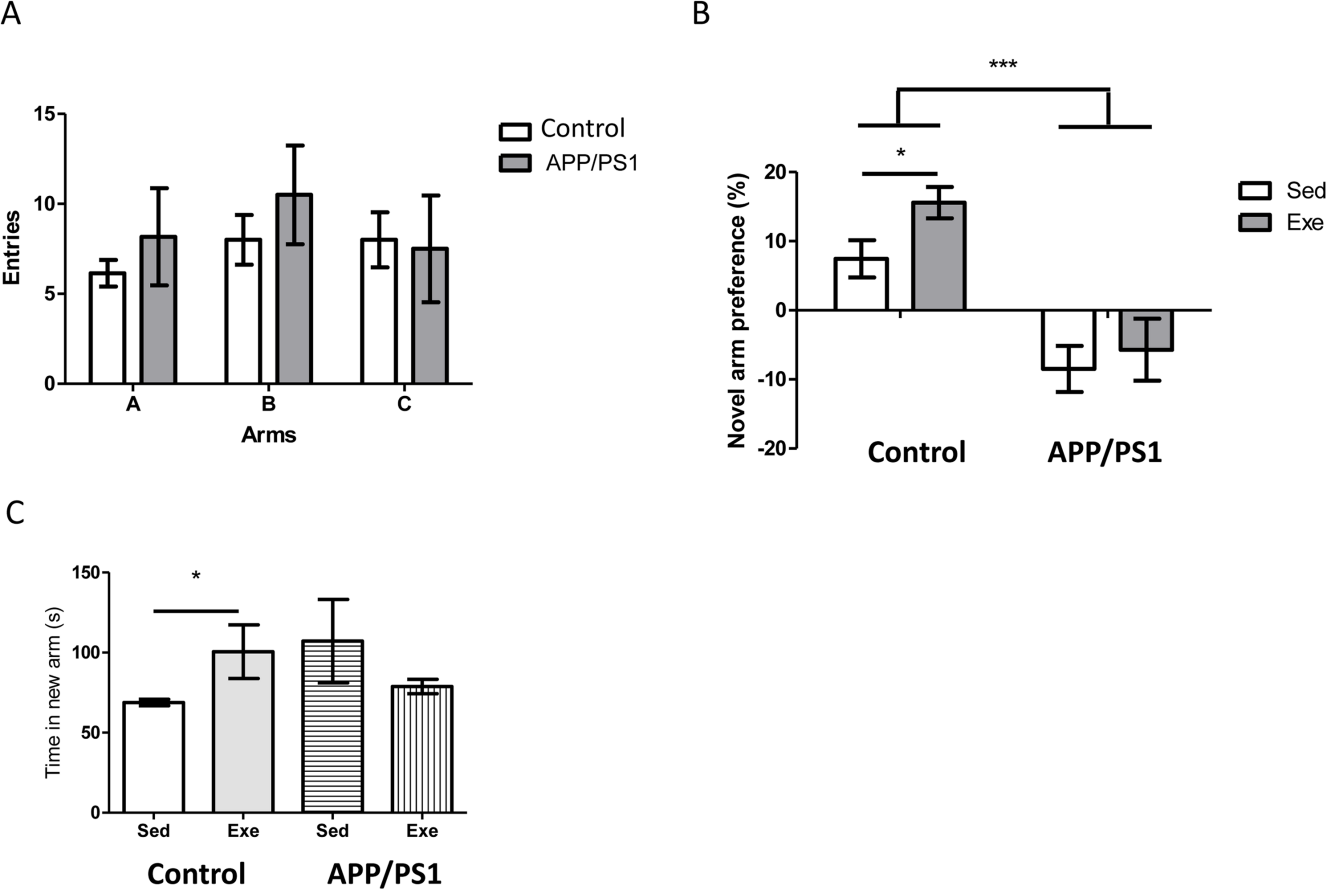
Y maze performance after exercise. **A,** Both control and APP/PS1 mice performed similar in the acquisition phase of the Y maze before exercise, showing a similar rate of entries in each of the three arms of the maze. **B,** However, while control mice significantly improved their performance after exercise in the Y maze, as determined by increased preference for the novel arm, APP/PS1 did not. **C,** Improved performance was corroborated by increased time spent in the novel arm only in control mice. Sed: sedentary; Exe: exercised (n = 6 per group; one-way ANOVA (exercise x genotype interaction): F = 28.78, df = 17; ***p<0.0001 control vs APP/PS1; post-hoc t-test: *p<0.05 vs sedentary).

We submitted another group of exercised mice (30 minutes) to a Y maze, a test that measures spatial working memory [20]. The sedentary group remained in the treadmill without running, for the same time. Under the relatively prolonged inter-trial conditions used in this test, control exercised mice entered the novel arm significantly more times and spent more time in it than the sedentary group, indicating that exercise enhanced working memory (Fig 2). Importantly, both sedentary and exercised mice were able to learn in the Y maze to the same extent when using a shorter inter-trial time (S1C Fig). However, while both control and APP/PS1 mice behaved similarly in the acquisition phase of the Y maze (Fig 2A), after exercise the latter did not improve their performance in the Y maze (Fig 2B and 2C).

## Discussion

In this proof-of-concept study we have taken advantage of the cognitive-promoting actions of exercise [21, 22], its association to cognitive fitness [7, 8], and its effects on brain activity [23], to show that normal mice improve cognitive skills in the Y maze after exercise. In parallel, mice showed exercise-induced changes in the ECG pattern, specifically in the theta wave. Conversely, APP/PS1 mice did not improve memory skills after exercise and did not show changes in theta after exercise. Collectively, these data indicate that exercise-induced increases in theta -an ECG frequency associated to attention [24], and memory [25], result in improved memory in healthy mice, but not in APP/PS1 mice, a well-established model of AD-like amyloidosis and cognitive deterioration.

Most studies on the effect of exercise on the EEG have reported increased activity in the alpha frequency band, which may reflect a state of decreased cortical activation in comparison with states with high cognitive activity that increase beta and gamma and reduce alpha frequency bands [26, 27]. However, new evidence suggests that regular physical activity can impact cortical function and facilitate plasticity. Aerobic exercise has the capacity to induce short-term neuroplasticity within the human motor cortex, as assessed through cortical circuits evoked by transcranial magnetic stimulation [28]. Our findings agree with these results indicating that the hippocampal theta rhythm -involved in rodents in many plasticity processes such as LTP [29], increases after exercise. APP/PS1 mice did not increase the power of the theta frequency band after exercise, suggesting that plasticity processes may be reduced. Accordingly, the EEG of AD patients shows an increase in slow frequencies (11–12).

Exercise is nowadays one of the most promoted approaches for prevention and treatment of Alzheimer´s disease [30]. In all cases, based on pre-clinical studies, chronic exercise of various intensities has been considered the appropriate regime. However, our results extend its utility as a potential diagnostic tool when used acutely. We consider that this wide utility of exercise is based on its ample neuroprotective actions, including increased brain perfusion, neurotrophic input and metabolic fitness [31–36]. All these beneficial actions, necessary to preserve normal levels of brain activity, may be partially diminished during aging, leading to gradual cognitive deterioration. We postulate that a bout of exercise unveils this gradual loss of exercise neuroprotection before cognitive deterioration develops. This is reflected in an abnormal pattern of brain activity recorded in the ECG in response to exercise; namely, no changes in theta activity.

Brain activity patterns previously reported using ECG in AD mice had shown general abnormalities [15] together with progressive changes along aging in all spectral frequencies [37], or specifically in theta and delta bands [38]. Other reports also indicate the existence of specific changes in theta and gamma activities [39–42], or in the whole ECG pattern [43]. These varied observations are probably due to the different types of AD mouse models used. However, collectively these data suggest that AD animal models exhibit altered cortical excitability and hippocampal dysrhythmicity. Our findings in APP/PS1 animals show cortical hyperexcitability because they exhibited a decrease of delta power and an increase in faster frequency bands at rest. These results are in agreement with the presence of seizure activity in AD animal models [44] and with the already mentioned fact that people with AD are more likely to develop epilepsy [45]. Our observations add to the growing potential translability of rodent studies to humans using electrophysiological recordings [46].

Indeed, with the idea of translating these observations to the clinical practice, our results indicate the feasibility of an easy-to-carry-out test to determine cognitive health in the general population based on accessible diagnostic tools. Similar translation studies from rodents [47] to humans [48] has proven successful for exercise as a protective measure against AD. Therefore, a simple diagnostic procedure derived from our observations and others [49], would consist in submitting test subjects to moderate exercise in conjunction with recording EEG activity before and after exercise. Individuals not showing changes after exercise in EEG activity will be categorized as “at-risk” and should undergo further testing. On-going studies in healthy volunteers and cognitively deteriorated subjects will help clarify the possible translation of this test to the clinic.

## Supporting information

S1 Fig **A,** Aβ immunostaining of control (WT) and APP/PS1 (AD) show the presence of small deposits (Aβ plaques) only in the latter. **B,** Time line of experimental procedure for Y maze plus exercise used in the experiments shown in Fig 2. **C,** Both sedentary (white bars) and exercised (grey bars) wild type mice learn the Y maze task as indicated by increased entries to the novel arm of the maze when using a shorter (60 min) inter-trial time (F = 15.774; df = 30; ***p<0.001; n = 9 per group).
(JPG)

## References

[1] Shah H, Albanese E, Duggan C, Rudan I, Langa KM, Carrillo MC, et al Research priorities to reduce the global burden of dementia by 2025. Lancet Neurol. 2016;15(12):1285–94. doi: 10.1016/S1474-4422(16)30235-6 27751558 10.1016/S1474-4422(16)30235-6

[2] Dubois B, Hampel H, Feldman HH, Scheltens P, Aisen P, Andrieu S, et al Preclinical Alzheimer's disease: Definition, natural history, and diagnostic criteria. Alzheimers Dement. 2016;12(3):292–323. doi: 10.1016/j.jalz.2016.02.002 27012484 10.1016/j.jalz.2016.02.002 PMC6417794

[3] Scheltens P, Blennow K, Breteler MM, de Strooper B, Frisoni GB, Salloway S, et al Alzheimer's disease. Lancet. 2016;388(10043):505–17. doi: 10.1016/S0140-6736(15)01124-1 26921134 10.1016/S0140-6736(15)01124-1

[4] Winter B, Breitenstein C, Mooren FC, Voelker K, Fobker M, Lechtermann A, et al High impact running improves learning. Neurobiol Learn Mem. 2007;87(4):597–609. doi: 10.1016/j.nlm.2006.11.003 17185007 10.1016/j.nlm.2006.11.003

[5] Kamijo K, Hayashi Y, Sakai T, Yahiro T, Tanaka K, Nishihira Y. Acute effects of aerobic exercise on cognitive function in older adults. J Gerontol B Psychol Sci Soc Sci. 2009;64(3):356–63. doi: 10.1093/geronb/gbp030 19363089 10.1093/geronb/gbp030

[6] Siette J, Reichelt AC, Westbrook RF. A bout of voluntary running enhances context conditioned fear, its extinction, and its reconsolidation. Learn Mem. 2014;21(2):73–81. PubMed Central PMCID: PMCPMC3895230. doi: 10.1101/lm.032557.113 24429425 10.1101/lm.032557.113 PMC3895230

[7] Muller J, Chan K, Myers JN. Association Between Exercise Capacity and Late Onset of Dementia, Alzheimer Disease, and Cognitive Impairment. Mayo Clin Proc. 2017;92(2):211–7. doi: 10.1016/j.mayocp.2016.10.020 28082018 10.1016/j.mayocp.2016.10.020

[8] Boyle PA, Buchman AS, Wilson RS, Leurgans SE, Bennett DA. Association of muscle strength with the risk of Alzheimer disease and the rate of cognitive decline in community-dwelling older persons. Arch Neurol. 2009;66(11):1339–44. PubMed Central PMCID: PMCPMC2838435. doi: 10.1001/archneurol.2009.240 19901164 10.1001/archneurol.2009.240 PMC2838435

[9] Enders H, Cortese F, Maurer C, Baltich J, Protzner AB, Nigg BM. Changes in cortical activity measured with EEG during a high-intensity cycling exercise. J Neurophysiol. 2016;115(1):379–88. PubMed Central PMCID: PMCPMC4760484. doi: 10.1152/jn.00497.2015 26538604 10.1152/jn.00497.2015 PMC4760484

[10] Vissing J, Andersen M, Diemer NH. Exercise-induced changes in local cerebral glucose utilization in the rat. J Cereb Blood Flow Metab. 1996;16(4):729–36. doi: 10.1097/00004647-199607000-00025 8964814 10.1097/00004647-199607000-00025

[11] Jeong J. EEG dynamics in patients with Alzheimer's disease. Clin Neurophysiol. 2004;115(7):1490–505. doi: 10.1016/j.clinph.2004.01.001 15203050 10.1016/j.clinph.2004.01.001

[12] van der HK, Vein AA, Reijntjes RH, Westendorp RG, Bollen EL, van Buchem MA, et al EEG correlates in the spectrum of cognitive decline. Clin Neurophysiol. 2007;118(9):1931–9. doi: 10.1016/j.clinph.2007.05.070 17604688 10.1016/j.clinph.2007.05.070

[13] Fonseca LC, Tedrus GM, Letro GH, Bossoni AS. Dementia, mild cognitive impairment and quantitative EEG in patients with Parkinson's disease. Clin EEG Neurosci. 2009;40(3):168–72. doi: 10.1177/155005940904000309 19715179 10.1177/155005940904000309

[14] Faigle R, Sutter R, Kaplan PW. Electroencephalography of encephalopathy in patients with endocrine and metabolic disorders. J Clin Neurophysiol. 2013;30(5):505–16. PubMed Central PMCID: PMCPMC3826953. doi: 10.1097/WNP.0b013e3182a73db9 24084183 10.1097/WNP.0b013e3182a73db9 PMC3826953

[15] Born HA, Kim JY, Savjani RR, Das P, Dabaghian YA, Guo Q, et al Genetic suppression of transgenic APP rescues Hypersynchronous network activity in a mouse model of Alzeimer's disease. J Neurosci. 2014;34(11):3826–40. PubMed Central PMCID: PMCPMC3951689. doi: 10.1523/JNEUROSCI.5171-13.2014 24623762 10.1523/JNEUROSCI.5171-13.2014 PMC3951689

[16] Fernandez AM, Jimenez S, Mecha M, Davila D, Guaza C, Vitorica J, et al Regulation of the phosphatase calcineurin by insulin-like growth factor I unveils a key role of astrocytes in Alzheimer's pathology. Mol Psychiatry. 2012;17(7):705–18. doi: 10.1038/mp.2011.128 22005929 10.1038/mp.2011.128

[17] Sarter M, Bodewitz G, Stephens DN. Attenuation of scopolamine-induced impairment of spontaneous alteration behaviour by antagonist but not inverse agonist and agonist beta-carbolines. Psychopharmacology (Berl). 1988;94(4):491–5. 2836875 10.1007/BF00212843

[18] Rivas J, Gaztelu JM, Garcia-Austt E. Changes in hippocampal cell discharge patterns and theta rhythm spectral properties as a function of walking velocity in the guinea pig. Exp Brain Res. 1996;108(1):113–8. 8721159 10.1007/BF00242908

[19] Mancuso M, Filosto M, Bosetti F, Ceravolo R, Rocchi A, Tognoni G, et al Decreased platelet cytochrome c oxidase activity is accompanied by increased blood lactate concentration during exercise in patients with Alzheimer disease. Exp Neurol. 2003;182(2):421–6. 12895452 10.1016/s0014-4886(03)00092-x

[20] Paul C-M, Magda G, Abel S. Spatial memory: Theoretical basis and comparative review on experimental methods in rodents. Behavioural Brain Research. 2009;203(2):151–64. doi: 10.1016/j.bbr.2009.05.022 19467271 10.1016/j.bbr.2009.05.022

[21] Byun K, Hyodo K, Suwabe K, Ochi G, Sakairi Y, Kato M, et al Positive effect of acute mild exercise on executive function via arousal-related prefrontal activations: an fNIRS study. Neuroimage. 2014;98:336–45. doi: 10.1016/j.neuroimage.2014.04.067 24799137 10.1016/j.neuroimage.2014.04.067

[22] Dimitrova J, Hogan M, Khader P, O'Hora D, Kilmartin L, Walsh JC, et al Comparing the effects of an acute bout of physical exercise with an acute bout of interactive mental and physical exercise on electrophysiology and executive functioning in younger and older adults. Aging Clin Exp Res. 2016. 10.1007/s40520-016-0683-6 27866346

[23] Brummer V, Schneider S, Abel T, Vogt T, Struder HK. Brain cortical activity is influenced by exercise mode and intensity 1. Med Sci Sports Exerc. 2011;43(10):1863–72. doi: 10.1249/MSS.0b013e3182172a6f 21364475 10.1249/MSS.0b013e3182172a6f

[24] Sellers KK, Yu C, Zhou ZC, Stitt I, Li Y, Radtke-Schuller S, et al Oscillatory Dynamics in the Frontoparietal Attention Network during Sustained Attention in the Ferret. Cell Rep. 2016;16(11):2864–74. PubMed Central PMCID: PMCPMC5024719. doi: 10.1016/j.celrep.2016.08.055 27626658 10.1016/j.celrep.2016.08.055 PMC5024719

[25] Klimesch W. EEG alpha and theta oscillations reflect cognitive and memory performance: a review and analysis. Brain Res Brain Res Rev. 1999;29(2–3):169–95. 10209231 10.1016/s0165-0173(98)00056-3

[26] Steriade M, Llinas RR. The functional states of the thalamus and the associated neuronal interplay. Physiol Rev. 1988;68(3):649–742. 2839857 10.1152/physrev.1988.68.3.649

[27] Crabbe JB, Dishman RK. Brain electrocortical activity during and after exercise: a quantitative synthesis. Psychophysiology. 2004;41(4):563–74. doi: 10.1111/j.1469-8986.2004.00176.x 15189479 10.1111/j.1469-8986.2004.00176.x

[28] Lulic T, El-Sayes J, Fassett HJ, Nelson AJ. Physical activity levels determine exercise-induced changes in brain excitability. PLoS One. 2017;12(3):e0173672 PubMed Central PMCID: PMCPMC5344515. doi: 10.1371/journal.pone.0173672 28278300 10.1371/journal.pone.0173672 PMC5344515

[29] Vertes RP. Hippocampal theta rhythm: a tag for short-term memory. Hippocampus. 2005;15(7):923–35. doi: 10.1002/hipo.20118 16149083 10.1002/hipo.20118

[30] Forbes D, Thiessen EJ, Blake CM, Forbes SC, Forbes S. Exercise programs for people with dementia. Cochrane Database Syst Rev. 2013;12:CD006489. 10.1002/14651858.CD006489.pub3 24302466

[31] Patten AR, Sickmann H, Hryciw BN, Kucharsky T, Parton R, Kernick A, et al Long-term exercise is needed to enhance synaptic plasticity in the hippocampus 1. Learn Mem. 2013;20(11):642–7. doi: 10.1101/lm.030635.113 24131795 10.1101/lm.030635.113

[32] Pedersen BK, Pedersen M, Krabbe KS, Bruunsgaard H, Matthews VB, Febbraio MA. Role of exercise-induced brain-derived neurotrophic factor production in the regulation of energy homeostasis in mammals. Exp Physiol. 2009;94(12):1153–60. doi: 10.1113/expphysiol.2009.048561 19748969 10.1113/expphysiol.2009.048561

[33] Ropelle ER, Flores MB, Cintra DE, Rocha GZ, Pauli JR, Morari J, et al IL-6 and IL-10 Anti-Inflammatory Activity Links Exercise to Hypothalamic Insulin and Leptin Sensitivity through IKK+¦ and ER Stress Inhibition. PLoS Biol. 2010;8(8):e1000465 doi: 10.1371/journal.pbio.1000465 20808781 10.1371/journal.pbio.1000465 PMC2927536

[34] Stranahan AM, Lee K, Becker KG, Zhang Y, Maudsley S, Martin B, et al Hippocampal gene expression patterns underlying the enhancement of memory by running in aged mice. Neurobiol Aging. 2008. 10.1016/j.neurobiolaging.2008.10.016 PMC2889222 19070401

[35] Takimoto M, Hamada T. Acute exercise increases brain region-specific expression of MCT1, MCT2, MCT4, GLUT1, and COX IV proteins. J Appl Physiol (1985). 2014;116(9):1238–50. 10.1152/japplphysiol.01288.2013 24610532

[36] Trejo JL, Carro E, Torres-Aleman I. Circulating insulin-like growth factor I mediates exercise-induced increases in the number of new neurons in the adult hippocampus. J Neurosci. 2001;21(5):1628–34. 11222653 10.1523/JNEUROSCI.21-05-01628.2001 PMC6762955

[37] Jyoti A, Plano A, Riedel G, Platt B. Progressive age-related changes in sleep and EEG profiles in the PLB1Triple mouse model of Alzheimer's disease. Neurobiol Aging. 2015;36(10):2768–84. doi: 10.1016/j.neurobiolaging.2015.07.001 26239174 10.1016/j.neurobiolaging.2015.07.001

[38] Jyoti A, Plano A, Riedel G, Platt B. EEG, activity, and sleep architecture in a transgenic AbetaPPswe/PSEN1A246E Alzheimer's disease mouse. J Alzheimers Dis. 2010;22(3):873–87. doi: 10.3233/JAD-2010-100879 20858963 10.3233/JAD-2010-100879

[39] Siwek ME, Muller R, Henseler C, Trog A, Lundt A, Wormuth C, et al Altered theta oscillations and aberrant cortical excitatory activity in the 5XFAD model of Alzheimer's disease. Neural Plast. 2015;2015:781731 PubMed Central PMCID: PMCPMC4398951. doi: 10.1155/2015/781731 25922768 10.1155/2015/781731 PMC4398951

[40] Ittner AA, Gladbach A, Bertz J, Suh LS, Ittner LM. p38 MAP kinase-mediated NMDA receptor-dependent suppression of hippocampal hypersynchronicity in a mouse model of Alzheimer's disease. Acta Neuropathol Commun. 2014;2:149 PubMed Central PMCID: PMCPMC4212118. doi: 10.1186/s40478-014-0149-z 25331068 10.1186/s40478-014-0149-z PMC4212118

[41] Goutagny R, Gu N, Cavanagh C, Jackson J, Chabot JG, Quirion R, et al Alterations in hippocampal network oscillations and theta-gamma coupling arise before Abeta overproduction in a mouse model of Alzheimer's disease. Eur J Neurosci. 2013;37(12):1896–902. doi: 10.1111/ejn.12233 23773058 10.1111/ejn.12233

[42] Rubio SE, Vega-Flores G, Martinez A, Bosch C, Perez-Mediavilla A, del Rio J, et al Accelerated aging of the GABAergic septohippocampal pathway and decreased hippocampal rhythms in a mouse model of Alzheimer's disease. FASEB J. 2012;26(11):4458–67. doi: 10.1096/fj.12-208413 22835830 10.1096/fj.12-208413

[43] Schneider F, Baldauf K, Wetzel W, Reymann KG. Behavioral and EEG changes in male 5xFAD mice. Physiol Behav. 2014;135:25–33. doi: 10.1016/j.physbeh.2014.05.041 24907698 10.1016/j.physbeh.2014.05.041

[44] Palop JJ, Chin J, Roberson ED, Wang J, Thwin MT, Bien-Ly N, et al Aberrant excitatory neuronal activity and compensatory remodeling of inhibitory hippocampal circuits in mouse models of Alzheimer's disease. Neuron. 2007;55(5):697–711. doi: 10.1016/j.neuron.2007.07.025 17785178 10.1016/j.neuron.2007.07.025 PMC8055171

[45] Born HA. Seizures in Alzheimer's disease. Neuroscience. 2015;286:251–63. doi: 10.1016/j.neuroscience.2014.11.051 25484360 10.1016/j.neuroscience.2014.11.051

[46] Platt B, Welch A, Riedel G. FDG–PET imaging, EEG and sleep phenotypes as translational biomarkers for research in Alzheimer's disease. Biochemical Society Transactions. 2011;39(4):874–80. doi: 10.1042/BST0390874 21787316 10.1042/BST0390874

[47] Huttenrauch M, Brauss A, Kurdakova A, Borgers H, Klinker F, Liebetanz D, et al Physical activity delays hippocampal neurodegeneration and rescues memory deficits in an Alzheimer disease mouse model. Transl Psychiatry. 2016;6:e800 doi: 10.1038/tp.2016.65 27138799 10.1038/tp.2016.65 PMC5070068

[48] Hoffmann K, Sobol NA, Frederiksen KS, Beyer N, Vogel A, Vestergaard K, et al Moderate-to-High Intensity Physical Exercise in Patients with Alzheimer's Disease: A Randomized Controlled Trial. J Alzheimers Dis. 2015;50(2):443–53. 10.3233/JAD-150817 26682695

[49] Hyodo K, Dan I, Kyutoku Y, Suwabe K, Byun K, Ochi G, et al The association between aerobic fitness and cognitive function in older men mediated by frontal lateralization 1. Neuroimage. 2016;125:291–300. doi: 10.1016/j.neuroimage.2015.09.062 26439424 10.1016/j.neuroimage.2015.09.062

