# Supplementary figures and images for: Acute exercise does not modify brain activity and memory performance in APP/PS1 mice
Source: PLoS One. 2017 May 22;12(5):e0178247. doi: 10.1371/journal.pone.0178247 (PMC5440045; doi:10.1371/journal.pone.0178247)

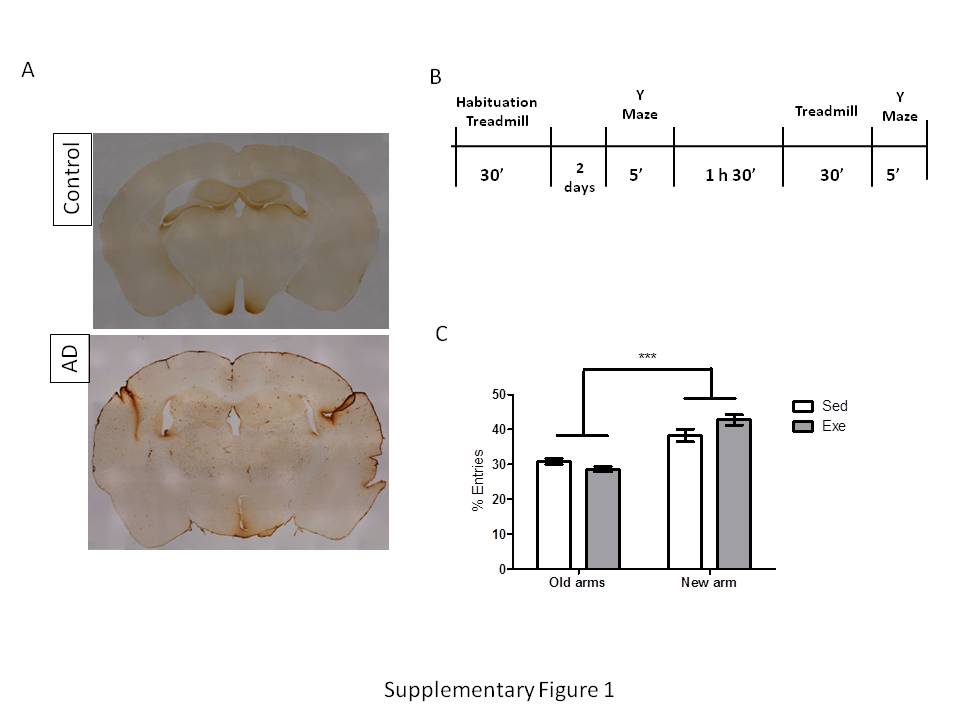

Supplement: S1 Fig — A, Aβ immunostaining of control (WT) and APP/PS1 (AD) show the presence of small deposits (Aβ plaques) only in the latter. B, Time line of experimental procedure for Y maze plus exercise used in the experiments shown in Fig 2. C, Both sedentary (white bars) and exercised (grey bars) wild type mice learn the Y maze task as indicated by increased entries to the novel arm of the maze when using a shorter (60 min) inter-trial time (F = 15.774; df = 30; ***p<0.001; n = 9 per group). (JPG) [file pone.0178247.s001.jpg]
